# Dr. H. N. Wadsworth

**Published:** 1896-10

**Authors:** 


### Bibliographical.

At his residence in Washington, D. C., October 9th, 1896, DR. H. N. WADSWORTH, aged seventy-six.

Hiram Nicholas Wadsworth was born in Burlington, Vermont, in February, 1819. He was of colonial stock, being a descendant of William Wadsworth, who landed in Boston in 1632, and was the father of the famous Captain Joseph Wadsworth, of Charter Oak fame, who, on the night of October 31st, 1687, seized and secreted the Charter of Connecticut when Sir Edwin Andros went to Hartford to take it by force from the colonists. Many of the family were conspicuous in the revolution and throughout the history of the country, in military, naval and civic life. Henry Wadsworth Longfellow was of the same ancestry.

Dr. Wadsworth spent a part of his earlier life in Ohio and in the State of New York, where he commenced the study of dentistry. He removed to the city of Washington in 1850 and practiced there continuously until 1893, when failing health compelled him to retire. He s'udied in the Baltimore College of Dental Surgery and graduated in

Though a man of by no means robust physique, but few practitioners have practiced longer than he did, and none more honorably. Few have succeeded in winning and retaining the confidence and patronage of so many prominent and distinguished clients. For many years he divided honors with the late Dr. Maynard, and numbered among his patients many of the most distinguished residents of the capital in diplomatic, civil and military circles, a number of whom remained in his hands until the very close of his professional career. This conspicuous success was not a mere matter of fortunate environment, nor was it wholly owing to the skill which he certainly possessed in a high degree; but, above all else, it was owing to his thorough honesty of purpose and his constant devotion to the highest professional ideal, coupled with all those finer and rarer qualities which mark the man of truly noble character. In his work he was careful and painstaking to an unusual degree. The true welfare of his patients was ever most prominently before him. He detested everything which savored in the least of quackery, and was, in short, a splendid example of the true professional man. To him, and a few others, dentistry is largely indebted for the somewhat unusual position and prestige which it enjoys in the Nation's capital.

Though an invalid for over three years, and for the greater part of that time confined continuously to his bed, he was bright and cheerful, retaining his interest in current topics and in professional progress, and was never known to complain. He became slowly weaker toward the last, and the end was easy and peaceful.

A widow and three daughters, two of whom are married, survive. A. W. S.

